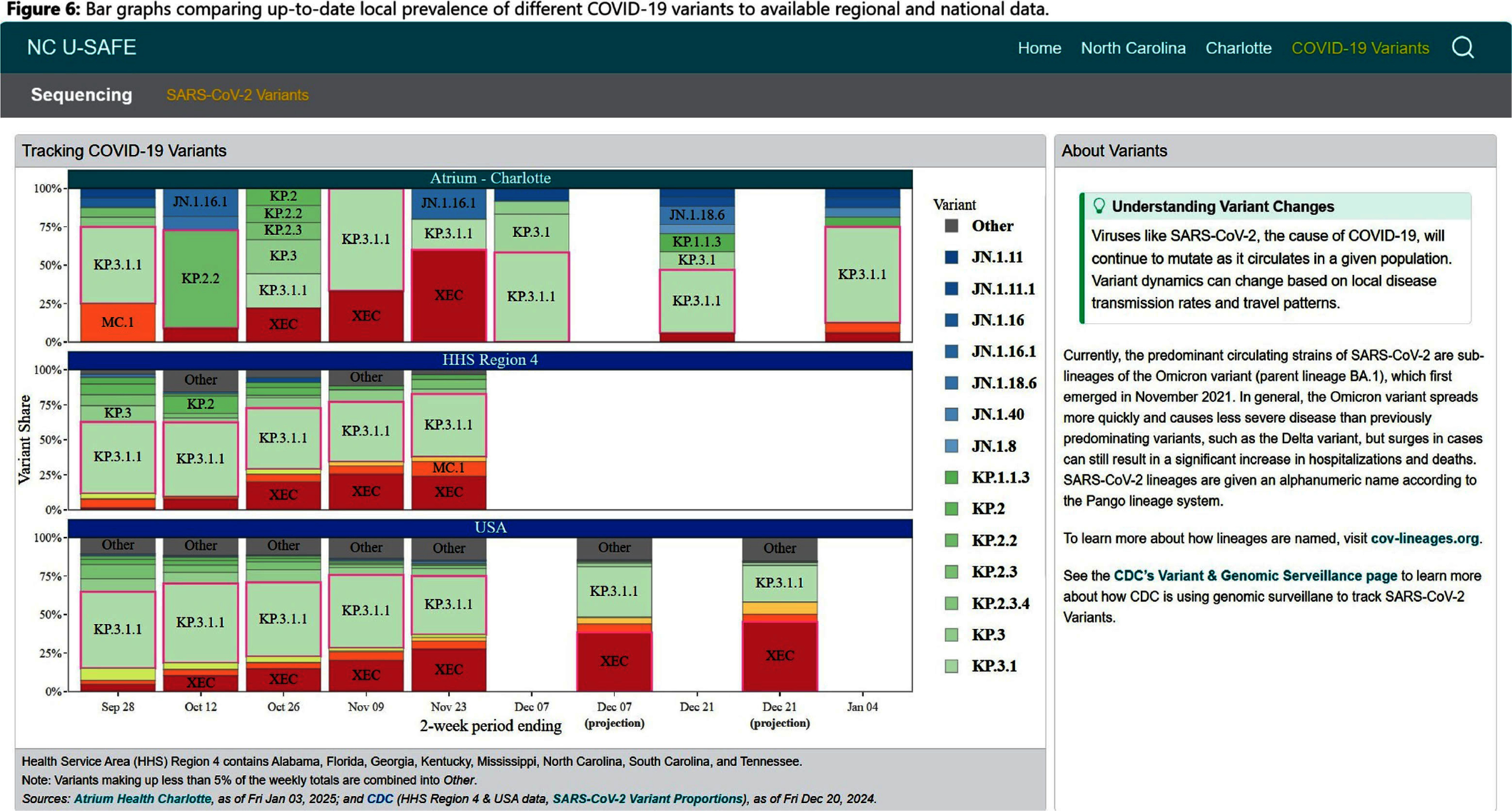# From Data to Decision: A Community-Engaged Respiratory Viral Surveillance and Forecasting Dashboard to Inform Public Health Measures

**DOI:** 10.1017/ash.2025.239

**Published:** 2025-09-24

**Authors:** Areej Bukhari, Jennifer Wenner, Amina Ahmed, Michael DeWitt

**Affiliations:** 1 Atrium Health Levine Children’s Hospital; 2 Section on Infectious Diseases, Wake Forest University School of Medicine; 3 Atrium Health; 4 Wake Forest University School of Medicine

## Abstract

**Background:** Public health communications during the COVID-19 pandemic demonstrated the value of web-based dashboards displaying trends in viral transmission to help inform individual and public health decision-making. However, these dashboards were often created without direct public engagement and lacked community-level data. We sought to bridge this gap by leveraging the data and resources of a large academic health center to create an extensible, dynamic respiratory viral trends dashboard incorporating feedback from community stakeholders. **Method:** Data on COVID-19, influenza and respiratory syncytial virus (RSV) testing and hospitalizations were programmatically collected from multiple sources, including the North Carolina Department of Health and Human Services, Centers for Disease Control and Prevention, and the healthcare system’s aggregated statistics and were presented on a web-based dashboard. Key statistics including test positivity rate, case counts, and trends were calculated. Projections were estimated using statistical and Bayesian semi-parametric models where sufficient data were available. Community stakeholders were identified through public health departments and the health facility’s community engagement department. The dashboard was presented to stakeholders quarterly over two years and electronic surveys solicited anonymous feedback after each meeting. A team of data scientists and infectious disease physicians served as the leads for dashboard development, meeting bi-weekly to address feedback and iterate on visual presentation. **Result:** The initial web-dashboard focused on COVID-19 variants and rapidly expanded to include RSV and influenza trends due to the predicted “tripledemic” in the 2022 respiratory viral season (Figures 1-7). The data were presented and allowed for comparison of national, state, and local disease dynamics and with data inputs updated weekly through an automated R program. Stakeholders included representatives from 6 county health departments, 2 colleges/universities, 2 community groups, and urgent care medical directors. A vast majority (17/18) felt that the knowledge gained was useful and not available to them from other sources, with 28% (5/18) indicating that the information presented informed practice, such as masking protocols or vaccine campaigns. The dashboard went through 3 major revisions in response to community stakeholder feedback. **Conclusion:** We describe the implementation of a community stakeholder-informed, web-based dashboard for the surveillance and forecasting of key respiratory viral pathogens utilizing national, regional and facility level data to help inform crucial decision-making for local health facilities and public health groups to curb transmission and spread. The framework of this platform can be built upon to include timely monitoring and public communication of the epidemiologic behavior of emerging pathogens in the community.

**Figure:**
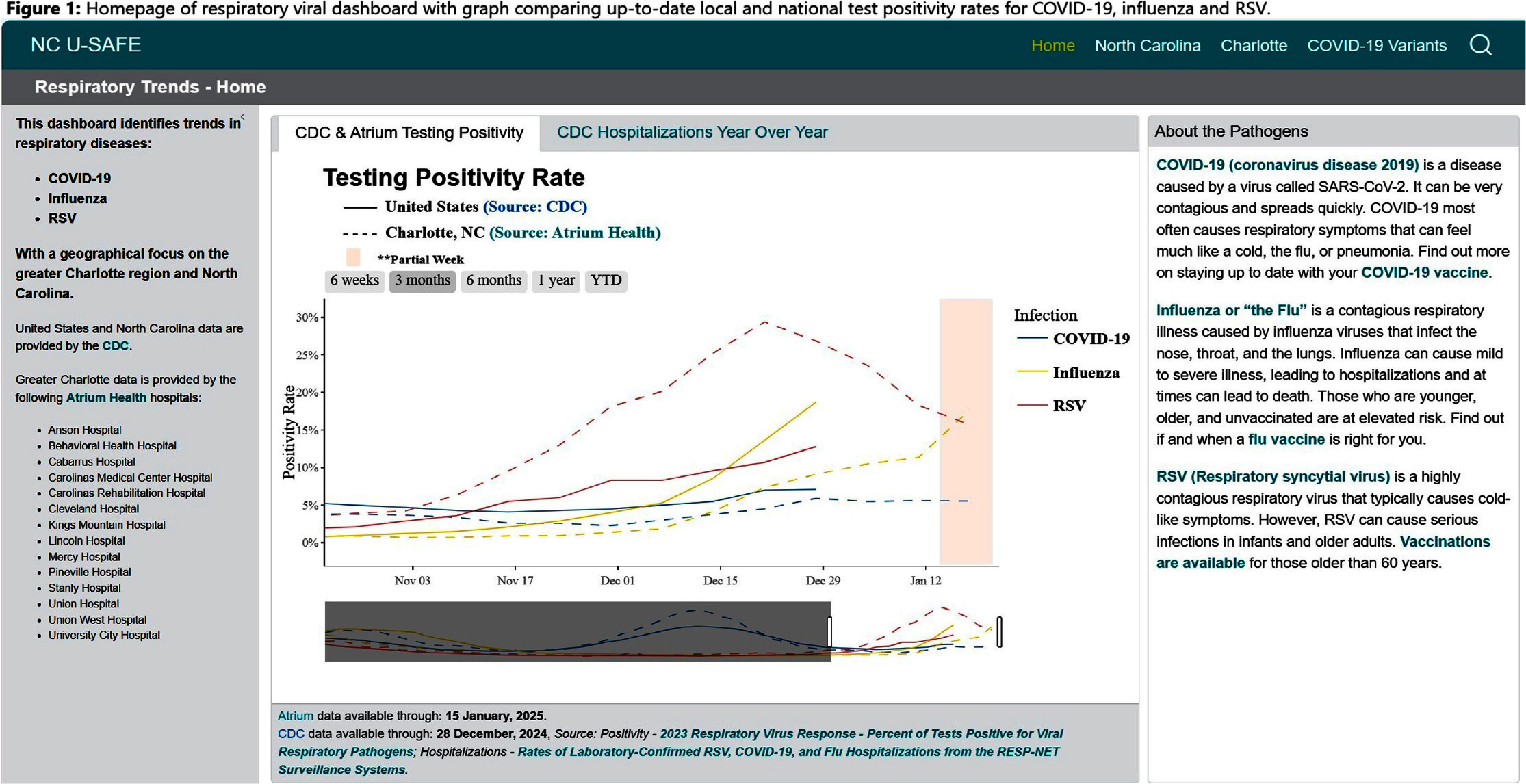

**Figure:**
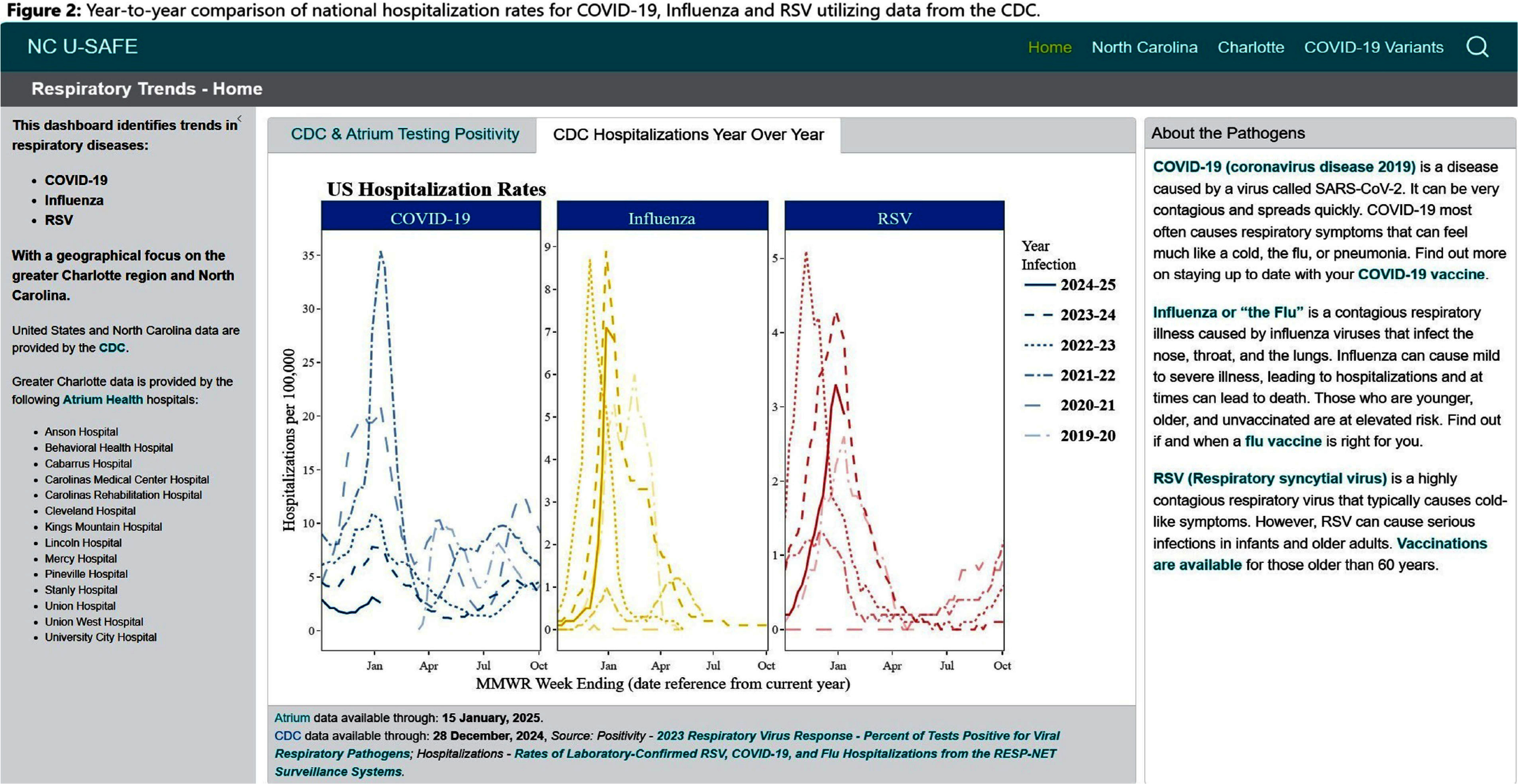

**Figure:**
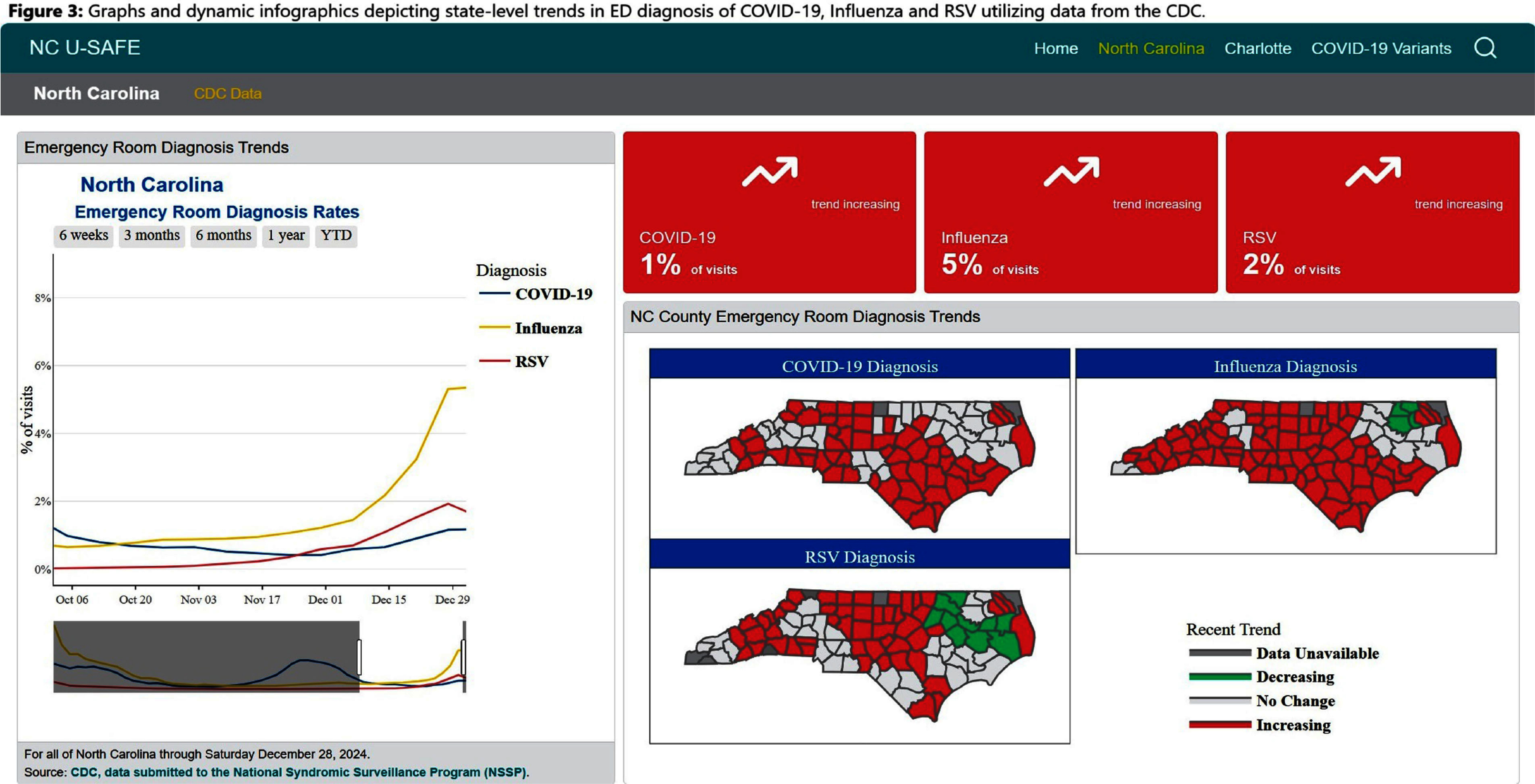

**Figure:**
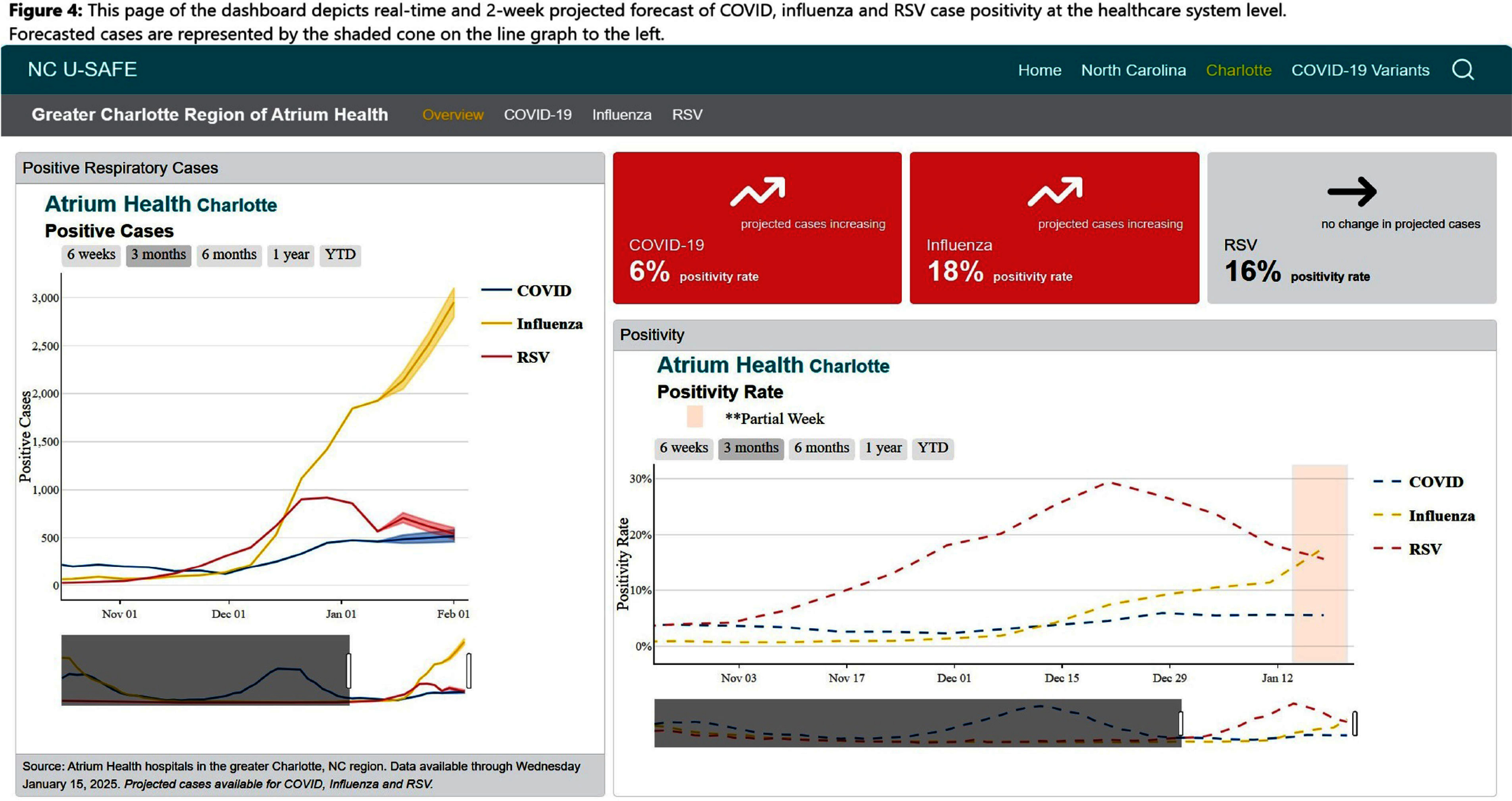

**Figure:**
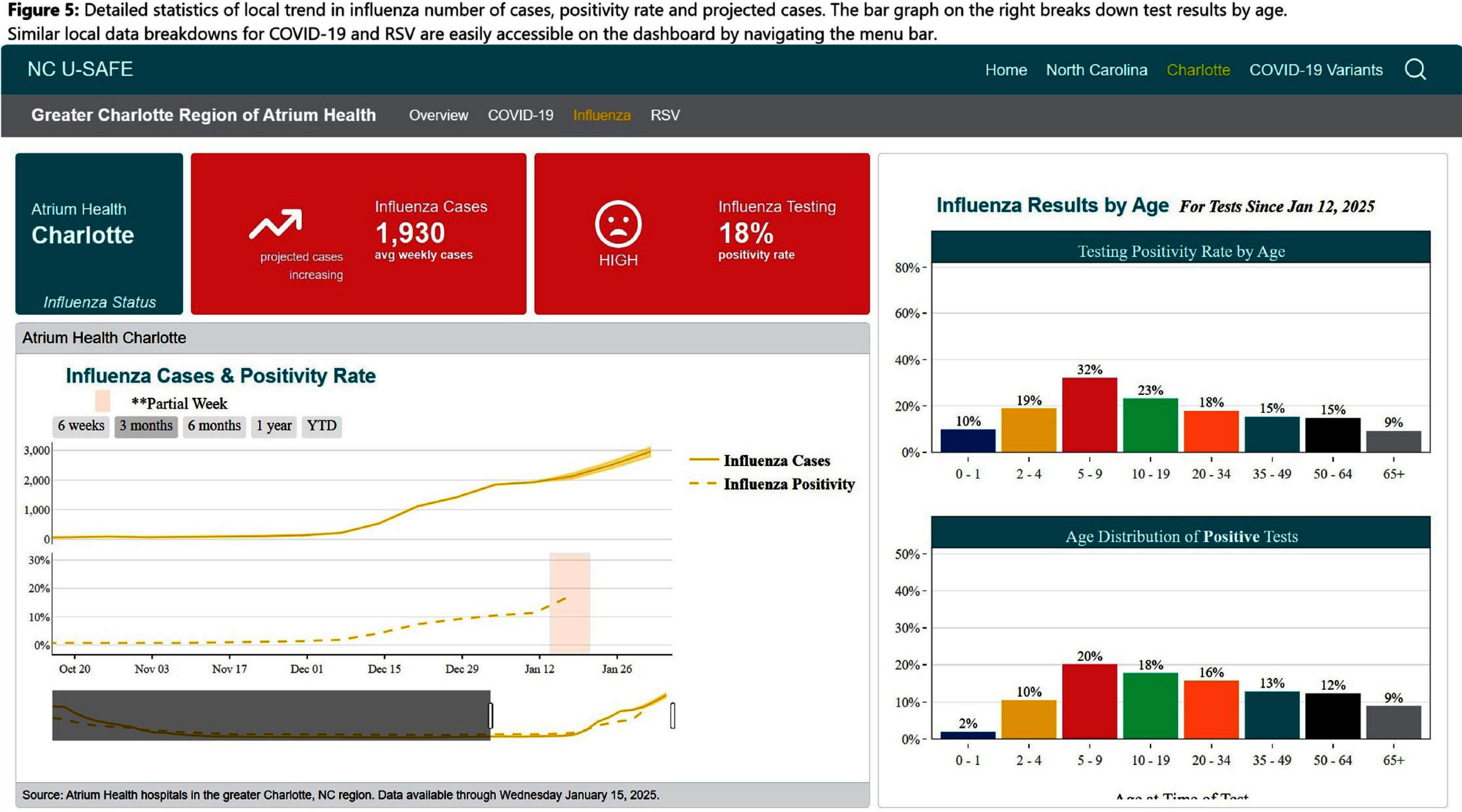

**Figure:**